# Electroretinography and contrast sensitivity, complementary translational biomarkers of sensory deficits in the visual system of individuals with fragile X syndrome

**DOI:** 10.1186/s11689-021-09375-0

**Published:** 2021-10-08

**Authors:** Olivier Perche, Fabien Lesne, Alain Patat, Susanne Raab, Roy Twyman, Robert H. Ring, Sylvain Briault

**Affiliations:** 1 Genetic Department, Centre Hospitalier Régional d’Orléans, Orléans, France; 2 UMR7355, Centre National de la Recherche Scientifique (CNRS), Orléans, France; 3 Experimental and Molecular Immunology and Neurogenetics, University of Orléans, Orléans, France; 4 Kaerus Bioscience Ltd., London, EC1Y 4YX UK; 5 Amron Neuroscience, LLC, Darby, MT USA; 6 Department of Pharmacology and Physiology, Drexel University College of Medicine, Philadelphia, PA USA

**Keywords:** Fragile X syndrome, FMR1, Electroretinography, Biomarker, ERG, Contrast sensitivity, Sensory hypersensitivity, Therapeutics

## Abstract

**Background:**

Disturbances in sensory function are an important clinical feature of neurodevelopmental disorders such as fragile X syndrome (FXS). Evidence also directly connects sensory abnormalities with the clinical expression of behavioral impairments in individuals with FXS; thus, positioning sensory function as a potential clinical target for the development of new therapeutics. Using electroretinography (ERG) and contrast sensitivity (CS), we previously reported the presence of sensory deficits in the visual system of the *Fmr1*^−/y^ genetic mouse model of FXS. The goals of the current study were two-folds: (1) to assess the feasibility of measuring ERG and CS as a biomarker of sensory deficits in individuals with FXS, and (2) to investigate whether the deficits revealed by ERG and CS in *Fmr1*^−/y^ mice translate to humans with FXS.

**Methods:**

Both ERG and CS were measured in a cohort of male individuals with FXS (*n* = 20, 18–45 years) and age-matched healthy controls (*n* = 20, 18–45 years). Under light-adapted conditions, and using both single flash and flicker (repeated train of flashes) stimulation protocols, retinal function was recorded from individual subjects using a portable, handheld, full-field flash ERG device (RETeval®, LKC Technologies Inc., Gaithersburg, MD, USA). CS was assessed in each subject using the LEA SYMBOLS® low-contrast test (Good-Lite, Elgin, IL, USA).

**Results:**

Data recording was successfully completed for ERG and assessment of CS in most individuals from both cohorts demonstrating the feasibility of these methods for use in the FXS population. Similar to previously reported findings from the *Fmr1*^*−/y*^ genetic mouse model, individuals with FXS were found to exhibit reduced b-wave and flicker amplitude in ERG and an impaired ability to discriminate contrasts compared to healthy controls.

**Conclusions:**

This study demonstrates the feasibility of using ERG and CS for assessing visual deficits in FXS and establishes the translational validity of the *Fmr1*^*−/y*^ mice phenotype to individuals with FXS. By including electrophysiological and functional readouts, the results of this study suggest the utility of both ERG and CS (ERG-CS) as complementary translational biomarkers for characterizing sensory abnormalities found in FXS, with potential applications to the clinical development of novel therapeutics that target sensory function abnormalities to treat core symptomatology in FXS.

**Trial registration:**

ID-RCB number 2019-A01015-52 registered on the 17 May 2019.

**Supplementary Information:**

The online version contains supplementary material available at 10.1186/s11689-021-09375-0.

## Background

Fragile X syndrome (FXS) (OMIM #300624) is a rare neurodevelopmental disorder caused by a trinucleotide (CGG) repeat expansion mutation in the promoter region of the fragile X mental retardation gene (*FMR1*, located at Xq27.3) [1]. The full mutation of *FMR1* (> 200 CGG repeats) is diagnostic for FXS and results in increased methylation of the *FMR1* promoter, silencing of the gene expression, and loss of its gene product (FMRP protein). FMRP is a multifunctional protein with a diverse range of known functions in the central nervous system (CNS) and some peripheral tissues. The loss of FMRP disrupts synaptic function, upsetting the balance of excitatory and inhibitory neurotransmission across the CNS and directly affecting symptom expression and severity in individuals with FXS [1–7].

Although behavioral, occupational and speech therapies, and off-label use of medicines developed for other CNS indications are available for the clinical management of FXS, there are currently no medical treatments approved for FXS [8]. Although there have been several clinical trials over the past few years evaluating potential treatments for cognitive and neurobehavioral symptoms of FXS, all have been unsuccessful [9, 10]. Factors contributing to trial failures likely include insufficient dosing and target engagement, lack of objective criteria for patient selection, and the use of clinical end points that are not sensitive enough to detect treatment response within the short duration of typical trials [11]. Furthermore, the measurement of deficits and therapeutic responses in individuals with intellectual disabilities comes with unique challenges that have further hampered drug development. There is an urgent need for biomarkers that can be used to objectively evaluate the biological pathways that underlie or contribute to the complex behavioral or cognitive outcomes being assessed in FXS trials [12].

A prominent feature of the FXS neurobehavioral phenotype is the disturbances of sensory processing including sensory hypersensitivity [13–16]. These disturbances commonly manifest as auditory hypersensitivity, impaired habituation to repeated sounds, reduced auditory attention [17, 18], tactile defensiveness [19], and significant visuospatial impairments [20, 21]. Interestingly, phenotypic similarities in some features between individuals with FXS and *Fmr1*^*−/y*^ mice (the genetic mouse model of FXS) suggest there are likely similar alterations in underlying sensory processing circuits across species that may provide a translational platform to understand underlying mechanisms and to also develop biomarkers for endophenotyping or for therapeutic testing [15]. Disrupted cytoarchitecture and signal processing of sensory circuits during early development may impair the ability to integrate sensory experiences leading to abnormal sensory circuit development, learning, cognitive skills, or anxiety that persist into adulthood [15]. Importantly, sensory disturbances also exacerbate the expression of intellectual disability (ID) and autistic features that are often sources of distress for individuals with FXS and their families.

Vision is a complex and highly developed important sensory system that is affected in FXS individuals. Altered visual processing in FXS could contribute to delayed sensory-motor features [22], impairments in neuropsychological tasks that require drawing skills and fine psychomotor coordination [23–25], and deficits in social emotion-recognition [26]. Studies have suggested that in FXS, disturbances in the visual sensory system correspond to a reduction in visual attention capacity that is associated with reduced sensitivity to contrasts, textures, and movements [20, 21, 27–30]. These impairments may be due to the synaptic immaturity observed in post-mortem labeling in the visual cortex [31]. Retinal neurons share the same neuroectodermal embryonic origins with cortical neurons [32, 33], and both neuron types express FMRP [34–37]. The retinal neural network shares similarities with other neural networks of the brain, in terms of connectivity [38, 39], but the retina’s accessibility allows for specific retinal signaling pathways to be probed and measured noninvasively with electroretinography (ERG). In the *Fmr1*^*−/y*^ genetic mouse model of FXS, absence of FMRP leads to impaired retinal signaling and visual perception in these mice, including altered retinal electrophysiological responses to stimuli as assessed by ERG [35, 40], deficits in contrast discrimination [41], reduction in the ability to translate a moving contrasted pattern [41], and impaired perspective perception [41]. *Fmr1*^*−/y*^ mice also exhibit deregulation of pre- and post-synaptic protein expression resulting in retinal neuron immaturity and synaptic destabilization similar to cortical neurons in *Fmr1*^*−/y*^ mice [31, 42].

ERG has been performed in other neurodevelopmental diseases and psychiatric disorders [43–46] and offers an opportunity to non-invasively explore visual system physiology in individuals with intellectual disability and complex pathologies such as FXS. Indeed, a recent systematic review has highlighted the growing use of ERG in psychiatric disorders [46]. For example, in individuals with schizophrenia, Hebert et al. [45] found smaller light-adapted (LA) ERG wave amplitudes with prolonged latencies, and in patients with depression, Hebert et al. [47] reported a delayed LA ERG response. Recently, ERG wave amplitudes had been used to differentiate between schizophrenia and bipolar disorder [48]. These findings suggest that ERG can reveal physiologic functional differences in the retina that might reflect synaptic transmission alterations in the CNS. Furthermore, the overlapping interactions of genes implicated in autism spectrum disorder (ASD), schizophrenia, or bipolar disorder [38, 39, 46, 49, 50] and the similarity between the deficits in ERG waves in these pathologies [43, 44, 48] suggests that ERG could potentially help to understand a diverse range of psychiatric disorders. In summary, the ERG is a non-invasive clinical tool that generates an objective output that can probe specific neural pathways, neurotransmitters, and their receptors in the retina. Hence, ERG may be an appropriate biomarker approach to probing underlying disease biology that could also facilitate therapeutic drug development, especially for agents that target neural signaling pathways and sensory systems [51, 52]. Advances in ERG recording technology have made the procedure considerably more user-friendly for neuro-impaired populations, especially the elimination of the need for corneal electrodes and mydriasis (pupil dilation).

The abnormalities in visual-system signaling and function found in the mouse model provide translational opportunities for better understanding their clinical relevance in FXS and for evaluating the potential utility of ERG and CS biomarker(s) for clinical development in FXS. The objectives of the current study were two-folds: first, to demonstrate that these evaluations can be done in individuals with FXS, and second, to investigate the translational validity of original observations in *Fmr1*^*−/y*^ mice to humans with FXS. Here, we report on the procedural details and feasibility for using ERG and CS protocols in clinical studies involving individuals with FXS, and we provide evidence of sensory deficits in the visual system using a combination of ERG recording and measures of CS. Together, these objective measurements offer complementary biomarkers of sensory dysfunction in the visual system of individuals with FXS, suggesting their potential application in the future discovery and development of therapeutics targeting the sensory abnormalities in FXS and related neurodevelopmental disorders.

## Methods

### Study and participants

All visual investigations were part of an exploratory clinical study named CLIBIOMAR FXS. This exploratory study of clinical and biological markers focused on the visual phenotype as assessed by ERG using ISCEV (International Society of Clinical Electrophysiology of Vision) recommendations, contrast sensitivity test using the LEA SYMBOLS® low-contrast test and Short Sensory Profile (SSP) questionnaire (French version) for other neurosensorial abnormalities. A total of 20 FXS subjects (male, 18–45 years of age), with a previously confirmed molecular genetic diagnosis of the full fragile X mental retardation mutation (≥ 200 CGG repetitions, fully methylated) (Table 1), and 20 male age-matched healthy control subjects (controls) were targeted to be enrolled in the study (Table 2). FXS subjects were identified from existing clinical populations in hospital files at the Genetics Laboratory of Orléans Regional Hospital Center (Orléans, France), the Biochemistry and Genetics Department of Angers University Hospital Center (Angers, France), the Clinical Genetics Department of Rennes University Hospital Center (Rennes, France), and two French FXS patient organizations (Mosaïque and Fragile X France, France) and invited to participate. Control subjects, recruited as a separate cohort through the Eurofins OPTIMED (Grenoble, France) database, were non-smokers and considered as healthy based on a comprehensive clinical assessment including history and a normal clinical examination. Participants in either group were excluded if there was a family history of ocular disease, strabismus, any history of epileptic seizures in the last year, or any history of head or brain trauma or pathology. To be included, control subjects had to be compliant to undergo ophthalmologic recordings. The level of ID was not an eligibility criterion for FXS subjects but a study investigator (SB) screened FXS volunteers for their ability to be neurobehaviorally compliant to ophthalmologic recordings.

**Table 1:** Detailed summary of subject characteristics—fragile X syndrome

Subject ID (FXS)			LA-ERG (Flash)			LA-ERG (Flicker)			LEA SYMBOLS® low-contrast				ABC-CFX (Total score)		SSP (Total score)
	Age	Iris^1^	Incl^2^	Exclusions	Eyes^3^	Incl^2^	Exclusions	Eyes^3^	Incl^2^	Exclusions	Eyes^3^	Incl^2^		Incl^2^	
**3-001**	39	1.4	Y	*-*	2	Y	*-*	2	Y	*-*	2	Y	58	Y	137
**3-002**	24	1.5	Y	*-*	2	Y	*-*	2	Y	*-*	2	Y	66	Y	112
**3-003**	20	1.6	Y	*-*	2	Y	*-*	2	Y	*-*	2	Y	58	Y	119
**3-004**	24	1.4	Y	*-*	2	Y	*-*	2	Y	*-*	2	Y	40	Y	154
**3-006**	36	1.2	N	Excluded (Electrode^4^)	0	N	Excluded (Electrode^4^)	0	Y	*-*	2	Y	29	Y	138
**3-007**	32	1.5	Y	*-*	2	Y	*-*	2	Y	*-*	2	Y	14	Y	157
**3-008**	21	-	N	Excluded (Behavior)	0	N	Excluded (Electrode^4^)	0	N	Excluded (Behavior^5^)	0	Y	42	Y	114
**3-009**	28	1.3	Y	*-*	2	Y	*-*	2	Y	*-*	2	Y	16	Y	166
**3-010**	40	1.4	Y	*-*	2	Y	*-*	2	Y	*-*	2	Y	44	Y	118
**3-011**	37	1.3	Y	*-*	2	Y	*-*	2	Y	*-*	2	Y	31	Y	129
**3-012**	27	1.5	N	Excluded (Electrode^4^)	0	N	Excluded (Electrode^4^)	0	Y	*-*	2	Y	10	Y	174
**3-013**	28	1.4	Y	*-*	2	Y	*-*	2	Y	*-*	2	Y	16	Y	163
**3-014**	26	1.4	Y	One eye (Electrode^4^)	1	Y	One eye (Electrode^4^)	1	N	Excluded (Behavior^5^)	0	Y	9	Y	130
**3-015**	18	1.2	N	Excluded (Electrode^4^)	0	Y	One eye (Electrode^4^)	1	N	Excluded (Behavior^5^)	0	Y	0	Y	157
**3-016**	28	1.4	Y	*-*	2	Y		2	Y	*-*	2	Y	86	Y	126
**3-017**	33	1.5	Y	One eye (Electrode^4^)	1	Y	One eye (Electrode^4^)	1	Y	*-*	2	Y	17	Y	116
**3-018**	29	1.7	Y	One eye (Electrode^4^)	1	Y		2	N	Excluded (Behavior^5^)	0	Y	48	Y	126
**3-019**	18	1.2	N	Excluded (Electrode^4^)	0	Y	*-*	2	Y	*-*	2	Y	77	Y	133
**3-020**	25	1.3	Y	-	2	Y	-	2	Y	*-*	2	Y	44	Y	155
**3-021**	29	1.4	Y	-	2	Y	-	2	Y	*-*	2	Y	64	Y	120
	29	29													
**Totals**	**28**	**1.4**	**17**		**26**	**17**		**30**	**16**		**32**	**20**		**20**	

^1^Iris color index

^2^Data included in specific analyses (Yes/No)

^3^Number of eyes recorded

^4^Data excluded due to electrode placement

^5^No data recorded due to behavioral issues

ABC-CFX score was assessed as previously described [48, 53]. The SSP score was based on Dunn classification [49]

**Table 2:** Detailed summary of subject--> characteristics—control participant without FXS

Subject ID (FXS)			LA-ERG (Flash)			LA-ERG (Flicker)			LEA SYMBOLS® low-contrast		
	Age	Iris^1^	Incl^2^	Exclusions	Eyes^3^	Incl^2^	Exclusions	Eyes^3^	Incl^2^	Exclusions	Eyes^3^
**4-001**	29	1.2	Y	*-*	2	Y	*-*	2	Y	*-*	2
**4-002**	32	1.3	Y	*-*	2	Y	*-*	2	Y	*-*	2
**4-003**	43	1.3	Y	*-*	2	Y	*-*	2	Y	*-*	2
**4-004**	25	1.2	Y	*-*	2	Y	*-*	2	Y	*-*	2
**4-005**	34	1.3	Y	*-*	2	Y	*-*	2	Y	*-*	2
**4-006**	33	1.2	Y	*-*	2	Y	*-*	2	Y	*-*	2
**4-007**	23	1.1	Y	*-*	2	Y	*-*	2	Y	*-*	2
**4-008**	22	1.3	Y	*-*	2	Y	*-*	2	Y	*-*	2
**4-009**	20	1.5	Y	*-*	2	Y	*-*	2	Y	*-*	2
**4-010**	31	1.2	Y	*-*	2	Y	*-*	2	Y	*-*	2
**4-011**	24	1.5	Y	*-*	2	Y	*-*	2	Y	*-*	2
**4-012**	42	1.5	Y	*-*	2	Y	*-*	2	Y	*-*	2
**4-014**	31	1.3	Y	*-*	2	Y	*-*	2	Y	*-*	2
**4-015**	29	1.3	Y	*-*	2	Y		2	Y	*-*	2
**4-016**	35	1.3	Y	*-*	2	Y		2	Y	*-*	2
**4-018**	29	-	N	Excluded (Electrode^4^)	0	N	Excluded (Electrode^4^)	0	Y	*-*	2
**4-019**	19	1.5	Y	*-*	2	Y	*-*	2	Y	*-*	2
**4-020**	22	1.4	Y	*-*	2	Y		2	Y	*-*	2
**4-021**	21	1.4	Y	*-*	2	Y	*-*	2	Y	*-*	2
**4-022**	32	1.4	Y	-	2	Y	-	2	Y	*-*	2
	29	29									
**Totals**	**29**	**1.3**	**17**		**38**	**17**		**38**	**16**		**40**

^1^Iris color index

^2^Data included in specific analyses (Yes/No)

^3^Number of eyes recorded

^4^Data excluded due to electrode placement

### Ethics approval and consent to participate

Written informed consent was obtained from control subjects. For subjects with FXS, the informed consent was provided by the legal representative (parents in all cases) prior to any study procedure. Eligibility of FXS subjects for the study included company of a caregiver able to answer to the behavioral scale questionnaire and having a high probability for compliance with all examinations and completion of the study. The study adhered to the tenets of the Declaration of Helsinki and were performed in accordance to the protocols approved by the institutional review board (CPP Est 1, Dijon, France), along with proper notifications made to the Agence Nationale de Sécurité du Médicament et des Produits de Santé (The French National Agency for Medicines and Health Products Safety or ANSM) prior to the conduct of the study.

Procedures involving healthy control volunteers were conducted at Eurofins OPTIMED’s clinical pharmacology unit (Grenoble, France), while procedures involving FXS subjects were conducted in the subject’s home in the presence of their primary caregiver (e.g., parent, family member) by the same investigators (SB, FL). After arrival at the FXS subject’s home, investigators spent time discussing the study with the patient and their caregiver and showed them the study equipment in order to build trust and confidence to ensure that the study was conducted smoothly without undue stress. For FXS subjects, evaluations were initiated with the administration of the Short Sensory Profile (SSP) questionnaire (see below) with the primary caregiver together with the FXS subject whenever possible. Thereafter, following light-adaptation (LA), ERG was assessed for both FXS and controls using the RETeval® device (described below). Under LA conditions, ERG recordings were first made using the single-flash protocol, followed by recordings under the LA-flicker protocol. After a 20-min rest period, contrast sensitivity testing was performed using the LEA SYMBOLS® low-contrast test (described below). ERG and CS testing were performed by the same investigators for both cohorts. The behavioral status of FXS subjects was assessed with the *Aberrant Behavior Checklist–Community in FXS: ABC-CFX* scale [53] and the SSP and ABC-CFX scoring was done only for the FXS cohort.

### Assessment criteria

#### Short Sensory Profile (SSP)

The *Short Sensory Profile* (SSP) is a standardized questionnaire that permits to clinicians and researchers to quickly gather information about sensory processing problems that interfere with functional performance in children [54–56]. It can be used to signal a potential difference in children’s responses and behaviors to commonly occurring sensory events as compared to children without disability. This scale has been shown to be adaptable for use in adults presenting intellectual disabilities [57]. The 38 items of the SSP are extracted from the 125-item-long version of the Sensory Profile, which is based on factor analyses and correlation studies from two samples of 117 and 1037 children with a variety of neurodevelopmental diagnoses [55]. The SSP consists of 7 sections: (1) tactile sensitivity, (7 items, maximal sub-score 35), (2) taste/smell sensitivity (4 items, maximal sub-score 20), (3) movement sensitivity (3 items, maximal sub-score 15), (4) under-responsive/seeks sensation (7 items, maximal sub-score 35), (5) auditory filtering (6 items, maximal sub-score 30), (6) low energy/weak (6 items, maximal sub-score 30), and (7) visual/auditory sensitivity (5 items, maximal sub-score 25). Sub-scores from all 7 sections are added to obtain a total score and together describes the subject`s overall sensory profile. The profile is compared against a control database comprised of a national sample of children without disabilities and allows a comparison to determine if the score is likely different from the control population. For each section, the sub-score belonged to a classification called “typical performance,” “probable difference,” or “definite difference” as compared to the control database sample.

#### Electroretinography (ERG)

ERGs were recorded in accordance with standards established by the International Society of Clinical Electrophysiology of Vision (ISCEV) under light-adapted (LA) conditions [58]. Note that in standard ERG recording sessions, ERGs are obtained in both scotopic (dark-adapted) and photopic (light-adapted) conditions. In a pilot effort to see if scotopic recordings can be performed in FXS individuals, high anxiety was generated by 20 min of dark-adaptation and thus scotopic ERG was not recorded on FXS subjects. LA-ERG recordings were obtained using RETeval® device (LKC Technologies Inc., Gaithersburg, MD, USA), a widely used medical device in clinical settings with appropriate regulatory clearances by the FDA and EMA in the USA (510(k) clearance) and EU (CE mark). It is a hand-held, portable stimulus and recording instrument designed for performing ERGs in pediatric individuals which has been validated in several studies generating results similar to the classical ERG device [59–61]. The instrument contains a normative reference range of values for ERG parameters [62, 63] and is ideally suited for the FXS study cohort with less compliant intellectually deficient individuals. Use of a single sticker recording electrode placed below the eye without requiring mydriasis greatly facilitated the challenges often encountered in clinical practice where corneal electrodes and mydriasis are required in classical ERG recordings (https://lkc.com/products/reteval-2/). ERG responses were digitally recorded from a self-adhesive skin electrode positioned below the lower eyelid using protocols that utilize constant retinal luminance without pupil dilation, which are described by the Troland unit (Td). In these protocols, the RETeval® device measures the pupil size in real time and continuously adjusts the flash luminance to deliver the targeted amount of light into the eye, regardless of the size of the pupil and according to the following formula: Troland = (pupil area in mm^2^) (luminance in cd/m^2^). It has been previously demonstrated that pupils do not need to be dilated to achieve consistent results [62, 64].

The participant was seated, and the skin electrode was placed 1–4 mm below the lower eyelid following skin preparation if required to reduce impedance to < 5 kΩ in accordance with the manufacturer’s instructions. The vertical and horizontal electrode position were recorded with a built-in infrared digital camera. Photographic images of each eye allowed post-analysis of electrode placement and fixation using a calibrated graticule. Electrode position can affect the amplitude of the ERG signal [65] and eyes for which the electrode position was > 4 mm were excluded. All images were inspected, and the position of the electrode measured with a weighting of 2 mm below the eye set at 0 in the statistical model. Since iris color can affect the ERG response, iris color index was used to weight the amplitudes according to iris pigmentation which can reduce the b-wave amplitude in heavily pigmented individuals [66]. Then, the participant was instructed to look steadily at a dim red LED located in the center of the Ganzfeld dome and to try to avoid blinking or eye movements. All recordings were performed under normal room lighting conditions, controlled by the RETeval® device. Retinal physiology was assessed using the Troland based ISCEV standard full-field white flash 3.0 cd s m^−2^ (85 Td.s) on a 30 cd s m^−2^ white background luminance (848 Td.s) at 2/s intervals made with 30 flashes averaged to generate the ‘single flash’ ERG waveform. This was followed by a 28.3-Hz series of repeated flashes (background luminance 848 Td) to generate the ‘flicker’ ERG. The right eye was always recorded first. Repeats of the recording were performed as required. ERG recordings typically lasted approximately 10 to 15 min to complete both eyes. Recordings were automatically stopped by the device if pupil tracking was lost (poor fixation), electrode impedance was > 5 kΩ (electrode placed improperly or came unstuck), or if pupil diameter was too small for the Troland protocol to provide the required flash strength for any specific retinal illuminance. In these cases, recordings were repeated up to twice more for each eye.

For this study, we compared the peak amplitudes and timings of LA-ERG a- and b-waves for the ISCEV standard light-adapted 3.0 flash (LA 3 cd s m^−2^, 2 Hz) and the 28.3 Hz flicker (LA 3 cd s m^−2^, 28.3 Hz). The ERG amplitude and time of the a- and b-waves were reported automatically by an algorithm and checked manually for accurate placement. If the a-wave amplitude was < 1 μV, the time and amplitude were ignored for that waveform. When repeated measurements were taken, the waveform with the largest b-wave amplitude was included. Raw data, video, and image of the electrode on the eye and iris color index were all exported for analysis using the RFF extractor version 2.9.4.1 (LKC Technologies Inc., Gaithersburg, MD, USA).

#### Contrast sensitivity assessment using the LEA SYMBOLS® low-contrast test

Contrast sensitivity (CS) was assessed with the LEA SYMBOLS® low-contrast test, which assesses an individual’s ability to discriminate between symbols (e.g., square, circle, house, apple) printed at a fixed size (10M) onto flashcards at 5 sequentially decreasing contrast levels (25%, 10%, 5%, 2.5%, 1.25%), and measured at three different distances (1, 3, and 5 m). This test allows detection of contrast sensitivity in a population with potential language and/or cognitive deficits commonly observed in patients with neurodevelopmental disorders like in FXS patients [67–69]. To reduce known effects of luminance variation on threshold values, all tests were performed under controlled lighting conditions of 120 lux verified by measurement with a luminometer (Data Recorder PCE-VDL16l® device) for all distances assessed. Prior to the start of the test investigators confirmed that FXS subjects were able to identify (verbally or by pointing out) the test symbols correctly using a flashcard with 100% contrast level. Two investigators were required to perform the test. One investigator stood at the specified distance in front of the subject with the flashcard and pointed to different symbols. Contrast cards were presented from high to low. The second investigator stood behind the test subject assuring that the subject’s vision was not disturbed (e.g., by light reflection of the flashcards) noting the score of the test subject at each contrast level evaluated. The evaluation was done for each eye separately while the other eye was covered by hand. Decreasing contrast levels were tested first at a distance of 1 m for the left eye while one hand covered the right eye. The procedure was repeated for the right eye while one hand covered the left eye. Thereafter, the test distance was increased to 3 m and the sequence started again. Ultimately, the distance was extended to 5 m. At each contrast level, at least three symbols on the flashcard were pointed to the subject. In case of successful identification of all test symbols, a score of “5” was noted prior moving on to the next lower contrast level. If the test subject failed to correctly name one or two symbols, a score of “4” or “3” was noted respectively. If a test subject failed three times at a contrast level, the test was stopped, either the other eye was assessed or proceeded to the next distance. The result of the evaluation is the score of correct answers for each contrast level at each distance (score between 0 and 5). A total success score was calculated based on the sum of averaged values for each subject across contrast levels at the three distances, thus providing a more global assessment of CS (maximum score of 25). Two scores were calculated for subjects during each assessment. First, the average number of correctly identified symbols between both eyes of each subject was recorded to provide a total success score for each contrast level and at each distance (each with a maximum score of 5.0).

#### Statistical methods

Analysis of descriptive and inferential statistics for ERG and CS assessments were performed using SAS® version V9.4 (Cary, NC, USA). Imputation methods were not used in this study to replace missing data. No power calculation was performed as this was an exploratory study. Comparison between individuals with FXS and matched controls were performed using an analysis of variance (ANOVA). The analysis consisted of the comparison of the mean parameters recorded in FXS and control subjects at 95% confidence intervals (CIs). Statistical tests were performed two-sided with an alpha risk fixed at 5%.

For ERG recording, each eye (left and right) of a test subject served as an independent data source. When duplicate measures were available for ERG for each eye at a specific time, the ERG with the highest b-wave was used as the ERG parameter for the analysis. Only ERGs whose vertical height was below or equal to 4 mm were kept for the analysis. For each subject, there are four ERG measures, ISCEV LA flash 85 Td. for both eyes and LA flicker 28.3 Hz from both eyes. Guided by the animal model results [35, 40], both b-wave single flash and flicker amplitude measurements were considered primary outcome parameters; secondary outcomes were the time to peak of the b-wave, the a-wave amplitude and time to peak, the ratio of the b-wave amplitude to the a-wave amplitude (b:a ratio), amplitude, and flicker implicit time (time to peak). The comparison between FXS subjects versus age-matched controls was made using a model including subject as random effect and study group, age, iris index, and electrode distance (vertical distance from lower end of the eye and the electrode) as fixed factors. Finally, to evaluate for potential influence of medications on the ERG waveform, a further sub-analysis was performed on the b-wave amplitude excluding those subjects on psychotropic medication(s), which may have effects on the ERG results, and thus comparing only FXS patients without medications at time of study to matched controls.

Data from contrast sensitivity testing were analyzed by an ANOVA performed on success rate, i.e., the score obtained on the LEA SYMBOLS® low-contrast sensitivity test at each distance (1, 3, and 5 m) and each contrast (1.25, 2.5, 5, 10, and 25%) for each eye. The model included study group (FXS/controls), eye (right and left), distance, contrast, and interaction terms as fixed factors and subject as random effect. Due to a significant interaction between group and distance and group*contrast, the study group effect was assessed separately for each level of the factor included in the corresponding interaction (for each distance and for each contrast). As a secondary analysis, this approach was replicated considering only the FXS subjects without any psychotropic medication(s) versus controls.

## Results

### Group characteristics and testing feasibility

Twenty FXS individuals were screened by one of the investigators (SB) and were identified to be potentially compliant with study procedures (Table 1), and control subjects from the Eurofins OPTIMED database were screened to identify compliant subjects (Table 2). A total of 20 male FXS subjects and 20 male age-matched control subjects of mainly Caucasian origin were enrolled to participate in the study. The mean age was 28.1 years for the FXS cohort and 28.8 years for the control cohort (Table 3). In the FXS cohort, 6 of the 20 subjects were taking one psychotropic medication (four on antipsychotics, two with selective serotonin reuptake inhibitors) on the day of testing. In the control group, participants were not taking any psychotropic medications during the course of the study. Although the ABC-CFX neurobehavioral assessment can vary over time, the scores obtained during the day of ophthalmic evaluation indicated a similarly affected group in the FXS cohort and comparable to scores found in FXS populations in therapeutics studies [54, 70] (Table 1).

**Table 3:** Summary characteristics of subjects participating in the study and in the retinal assessment using the RETeval*®* device

**Study participant information**^**1**^	**FXS**	**Control**
Age^a^	**28.10** (6.53) [25.04-31.16]	**28.80** (6.83) [25.60-32.00]
Male gender^b^	**20** (100%)	**20** (100%)
Ethnicity^c^	**20** (100%) Caucasian	**18** (90%)^d^ Caucasian
**Short Sensory Profile** (SSP)^e^		
Subcore 1: Tactile sensitivity	26.05 (4.84) - Probable difference	-
Subcore 2: Taste/smell senstivity	18.15 (1.90) - Typical performance	-
Subcore 3: Movement sensitivity	**9.95 (4.27) - Definite difference**	-
Subcore 4: Under-responsive/seeks sensation	28.2 (4.85) - Typical performance	-
Subcore 5: Auditory filtering	**17.45 (4.16) - Definite difference**	-
Subcore 6: Low energy/weak	**18.65 (5.35) - Definite difference**	-
Subcore 7: Visual/auditory sensitivity	16.2 (6.07) - Probable difference	-
**Total Score**	**137.2 (19.52) - Definite difference**	-
**Participant information for Retinal assessments**^**1**^	**FXS**	**Control**
Age^a^	**28.12** (6.60) [24.72-31.51]	**28.79** (7.02) [25.41-32.17]
**n** (eyes) LA-ERG single flash^f^	**26**	**38**
**n** (eyes) LA-ERG flickers stimulation^f^	**30**	**38**
Iris/Pupil Ratio^a^	**1.38** (0.12) [1.35-1.43]	**1.33** (0.11) [1.30-1.37]
Electrodes vertical position^g^		
0 mm	0%	0%
1 mm	6.25%	10.52%
2 mm	18.75%	55.26%
3 mm	46.875%	26.31%
4 mm	28.125%	7.89%

^1^Characteristics are broken out into FXS and healthy control cohorts

^a^Data reported as mean (SD) [95% confidence level]

^b^Data are reported as *n* (%)

^c^Data are reported as *n* (%)

^d^Other two subjects were of Indian and African background

^e^Assessment scores (subscores and total) for Short Sensory Profile are reported here as the mean (SD) along with each corresponding Dunn Classification [49]

^f^Number of eyes

^g^Data are reported as percentage (%)

Within the FXS cohort, 15 of 20 subjects (75%) were able to complete the single flash ERG in at least one eye, and 12 of 20 had recordings successfully completed in both eyes (Table 1). The three subjects whose behavior (*n* = 1) or electrode placement (*n* = 2) interfered with the successful recording were the same three subjects where flash ERG could not be recorded. Similarly, 14 of 20 subjects successfully completed flicker ERG in both eyes, and 17 of 20 completed flicker in at least 1 eye (85%). For control subjects, 19 of 20 (95%) provided usable ERG data for both eyes and both single flash and flicker ERG (Table 2). One control cohort participant was excluded from ERG recording because of anatomical and/or physiological features that disabled detection of the suitable electrical signal by the electrode. For the LEA SYMBOLS® low-contrast test, 16 of 20 subjects (80%) in the FXS cohort were able to supply CS data for both eyes (Table 1). Four subjects could not provide data due to behavior-related reasons. All control subjects were able to provide CS data (Table 2). Taken together as a measure of visual system performance, an adequate ERG recording or CS assessment could be obtained for at least 1 eye in 19 of 20 FXS subjects (95%). For the SSP assessment, all 20 FXS subjects contributed data (Table 1).

Six subjects were taking psychotropic medication(s), resulting in a subset of 14 subjects that were not taking medications and were considered for the subset analysis in medication free subjects. Note that some subjects in this subset could not provide data for analysis for either one or both eyes.

### Short Sensory Profile scale (SSP)

The SSP scores of the FXS cohort highlighted a likely “probable” or “definite difference” alteration of sensory processing in several of the seven sensory subscores and a “definite difference” in the overall total score compared to the control database (Table 3). Among the subscales, the “definitive difference” classifications were “auditory filtering,” “movement sensitivity”, and “low energy/weak”); the “probable difference” classifications were “tactile sensitivity” and “visual/auditory sensitivity,” and the “typical performance” were “taste/smell sensitivity and “under-responsive/seeks sensation” (Table 3).

### Retinal physiology assessed by ERG

Together, the study presents a total of 26 FXS eyes and 38 control eyes for the LA single flash investigation, and a total of 30 FXS eyes and 38 control eyes for the LA flicker stimulation (Table 3). Using the RETeval® device, the iris color index was computed as a ratio of the 25th centile gray values obtained from two 1-mm-line segments centered vertically from the pupil margin to the 25th centile gray scale values of the pupil diameter. The overlapping confidence intervals indicated a similar iris color index between the FXS [1.35–1.43] and controls [1.30–1.37] groups, thus allowing direct comparison of ERG data (Table 3).

**Fig. 1:**
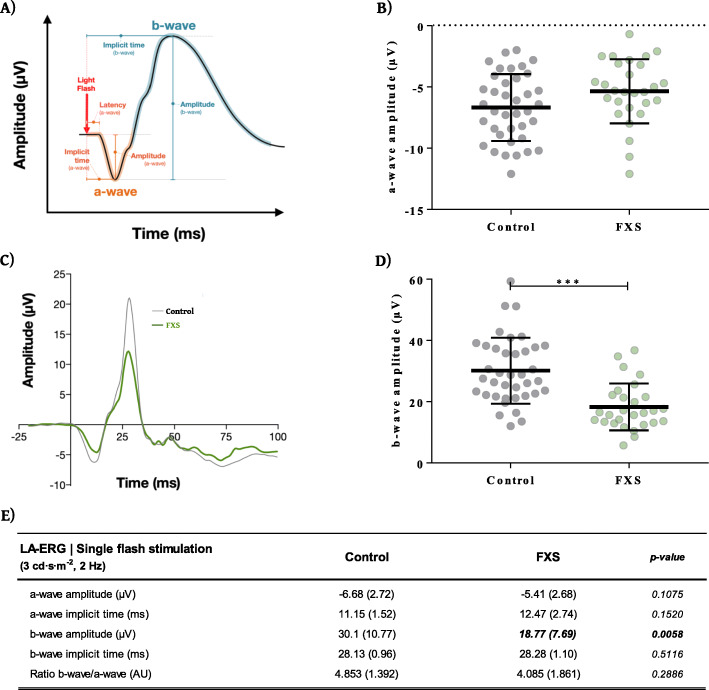
Summary data from ERG single flash stimulation. **A** Example diagrammatic view of the canonical waveform shape of a typical ERG recording, highlighting the key waveform components captured and measured in this study (a-wave and b-wave amplitudes, implicit times, and latency). Summary of LA-ERG (**B**) a-wave and (**C**, **D**) b-wave amplitude recordings measured in response to stimulation with single flash light protocol reveals a significant 37.6% decrease in b-wave amplitude. **C** ERG waveform traces summarizing the mean comparison of ERG recordings from both FXS (green) and healthy study control (black) cohorts in response to stimulation with the ISCEV standard LA single flash protocol. **E** Summary data table for LA-ERG single flash protocol

ERG waveform parameters that were measured are shown for single flash ERG in Fig. 1a. ISCEV standard LA single flash (3 cd s m^−2^, 2 Hz) analysis revealed a highly significant group effect for the b-wave amplitude [*F*(1, 36.1) = 8.59, *p* = 0.0058] whereas age [*F*(1, 30.3) = 1.23, *p* = 0.2764], eye [*F*(1, 31) = 0.01, *p* = 0.9253], iris index [*F*(1,50.5) = 0.25, *p* = 0.6177], and electrode distance [*F*(1, 48.8) = 1.25, *p* = 0.2691] had no effect. These results show significantly different electrophysiological parameters between FXS and control subjects. The mean b-wave amplitude of the FXS subjects was significantly decreased (*p* = 0.0058) compared to the control group (Fig. 1c–e). Analysis of the FXS a-wave revealed a trend but not a significant difference from the control group [*F*(1, 33.1) = 2.74, *p* = 0.1075], age [*F*(1, 26.5) = 0.09, *p* = 0.7610], eye [*F*(1, 30) = 0.02, *p* = 0.9002], iris index [*F*(1, 55.5) = 0.05, *p* = 0.8251], or electrode distance [*F*(1, 57.0) = 0.29, *p* = 0.5909] (Fig. 1b). Similarly, no difference was observed for a- or b-waves implicit times (Fig. 1e). In summary, the LA single flash stimulation indicated that the retinal response to light is altered in the FXS cohort with a 37.6% decrease in b-wave amplitude compared to age-matched control subjects. In addition, the sub-analysis performed on the single flash b-wave amplitudes using data excluding those FXS subjects on psychotropic medications (exclusion of *n* = 4 eyes among the 26 eyes of the full cohort) compared to the matched control showed a significantly decreased (*p* = 0.0084) b-wave amplitude [19.29 ± 7.45 μV] compared to controls [30.10 ± 10.77 μV] which was similar to that observed for the full cohort.

**Fig. 2:**
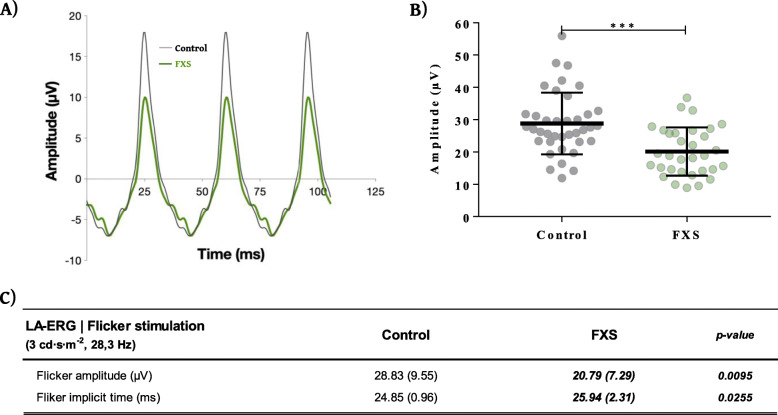
Summary data from ERG Flicker stimulation. **A** Representative raw waveform trace of LA-ERGs produced by flicker light stimulation protocol from an individual with FXS (green) and healthy study control (black) in response to a 28.3-Hz train of repeated flashes of light (flickers protocol). **B**, **C** Comparison of LA-ERG waveform parameters recorded from FXS and healthy study control cohorts in response to 28.3 Hz flicker stimulation reveals a significant 27.5% reduction in LA-ERG amplitude in individuals with FXS healthy study control. **C** A significant 4.8% reduction in LA-ERG flicker implicit time was also observed in the FXS cohort when compared to controls.

ERG flicker stimulation amplitude was measured from peak to trough of the waveform response and flicker implicit time was measured from the light flash to the peak of the first flicker waveform response. ISCEV standard LA flicker stimulation (3 cd s m^−2^, 28.3 Hz) statistical analysis revealed a significant group effect for flicker amplitude [*F*(1, 37.6) = 7.46, *p* = 0.0095], iris index [*F*(1, 59.8) = 5.20, *p* = 0.0261], and electrode distance [*F*(1, 56.8) = 4.19, *p* = 0.0452] whereas age [*F*(1, 33.7) = 2.43, *p* = 0.1282] and eye [F(1, 33.3) = 0.02, *p* = 0.9025] had no impact. For flicker implicit time, only a group effect was observed [*F*(1, 30.0) = 2.34, *p* = 0.0255]. FXS patients’ flicker response was significantly impaired as shown by a decreased amplitude (*p* = 0.0095) and increased implicit time (*p* = 0.0255) compared to the control group (Fig. 2a–c). In summary, the LA flicker stimulation showed a 27.5% decrease in flicker amplitude and 4.8% increase in flicker implicit time (Fig. 2c).

The results of the current study demonstrate that (1) the ERG methods can be successfully administered in a home setting to individuals with FXS, and (2) that data collected from individuals with FXS using ERG reveal a clear deficit in visual neural signaling not present in age-matched healthy controls.

### Contrast sensitivity assessment using the LEA SYMBOLS® low-contrast test

**Fig. 3:**
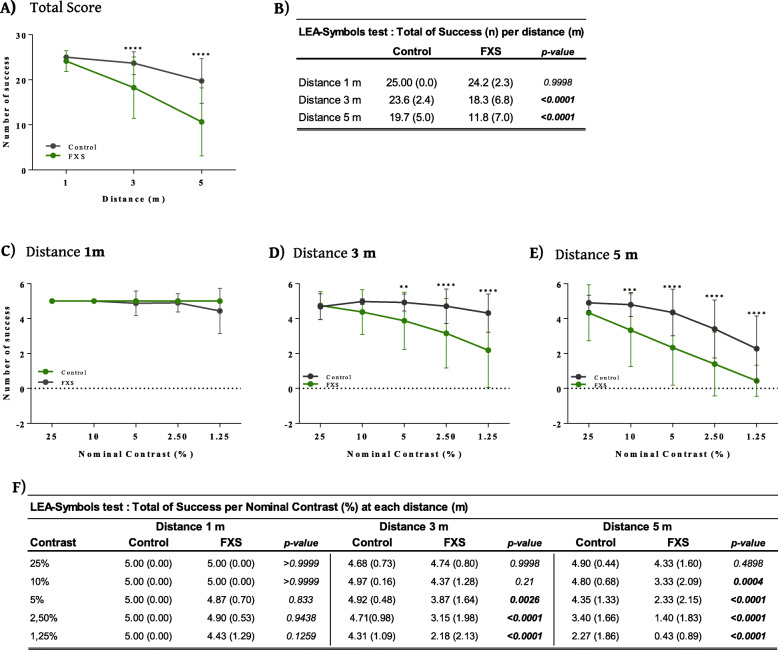
Summary data from LEA SYMBOLS® low-contrast sensitivity test. **A**, **B** When calculating total number of successes to discriminate symbols (25 maximum) for each of the three viewing distances (1, 3, and 5 m) significant reductions in FXS scores were observed at 3 m and 5 m. **C** Although no difference was observed at 1 m of distance, **D** FXS subjects exhibited significantly lower contrast sensitivity compared to healthy study controls at nominal contrast values of < 5% for 3 m of distance and **E** at < 10% nominal contrast at 5 m of distance. **F** Summary data table for LEA SYMBOLS® low-contrast sensitivity test

Results were available for both eyes for 16 FXS subjects (80%) and 20% of the data loss was due to procedural non-compliance (behavioral issues). Statistical analysis of the total success score (sum of success to discriminate the symbols at three distances) revealed a significant group effect [*F*(1, 34.3) = 18.99, *p* = 0.0001] and distance effect [*F*(1, 176) = 191.82, *p* < 0.0001] whereas there was no eye effect [*F*(1, 174) = 0.99, *p* = 0.3202]. These data indicate that both FXS and control subjects showed a significant decrease in total success score with increased distance and that FXS subjects were significantly different from controls (Fig. 3a, b). The contrast discrimination ability of the FXS subjects was lower compared to the control group especially at 3 m (*p* < 0.0001) and 5 m (*p* < 0.0001) of distance (Fig. 3a, b).

As the contrast discrimination performance was decreased in the FXS cohort, contrast sensitivity threshold can be determined for each distance. In this way, the performance of FXS and control groups was investigated for each nominal contrast tested (1.25, 2.5, 5, 10, and 25%) for each distance (1, 3, and 5 m) (Fig. 3c–f). Statistical analysis revealed a significant effect between groups [*F*(1, 35.0) = 7.38, *p* = 0.0102] and nominal contrast conditions [*F*(1, 328) = 88.69, *p* < 0.0001], whereas the eye condition [*F*(1, 328) = 1.60, *p* = 0.2064] had no effect. These data indicated that nominal contrast sensitivity for each distance is significantly different (*p* = 0.0102) in FXS subjects compared to the control cohort (Fig. 3f). Although no between-group difference was observed at the 1 m distance at any nominal contrast level (Fig. 3c, f), at 3 m FXS subjects scored significantly lower (*p* = 0.0026) compared to the control group beginning at the 5% level of nominal contrast and scored increasingly worse at the contrast levels were decreased. At 5 m distance, FXS subjects scored significantly lower (*p* = 0.0004) compared to the control group beginning at the 10% level of nominal contrast and lower contrast levels (Fig. 3d–f). In summary, the FXS cohort presented a significantly lower ability to discriminate contrast compared to the control group especially when the visual system is stressed by lower contrast situations and greater distance from the visual target.

The sub-analysis performed on the total success score using data excluding those subjects on psychotropic medications (exclusion of *n* = 6 eyes among the 32 eyes of the full cohort) compared to the matched control showed a similar profile as observed with the full FXS cohort. FXS without medication present a significantly decreased (*p* = 0.0004) total success score.

The results of the current study clearly demonstrate that (1) the LEA SYMBOLS® low-contrast test of CS can be successfully administered in a home setting to individuals with FXS, and (2) that data collected from individuals with FXS using this test indicate a significant deficit in contrast sensitivity compared to age-matched healthy controls.

## Discussion

FXS, the most common inherited form of intellectual disability associated with autistic-like behaviors, is characterized by cerebral sensory processing deficits and sensory abnormalities, which are at the center of the behavioral phenotype. Among all sensory abnormalities, the visual pathway seems to be particularly affected in individuals with FXS [21, 30]. In absence of FMRP, both retinal and cerebral structures of the visual pathway are impaired, suggesting that perception and integration of visual stimuli are altered [35, 40, 41]. Based on our original findings in *Fmr1*^*−/y*^ mice, the validated mouse model for FXS, we further investigated whether the deficits in ERG and CS were translatable to humans with FXS and could be potentially used as quantitative, objective biomarkers of sensory abnormalities. According to the Outcome Measures Working Group convened by the National Institutes of Health the absence of direct-observation measures or validated biomarkers was a key issue in clinical trials for treatment of FXS [71]. Two critical questions for understanding the clinical relevance of ERG and CS measurements in FXS and their potential utility as biomarker(s) in future treatment development were raised in this study. Firstly, we aimed to demonstrate that these visual evaluations can be performed in individuals with FXS, and secondly, we wanted to investigate whether the abnormal retinal physiology and visual function observations in *Fmr1*^*−/y*^ mice would translate to patients.

Performing clinical investigations in patients with ID and/or autistic behavior remains challenging, especially when these individuals’ medical experiences can provoke anxiety, exacerbate stress, and increase difficulties with performing clinical examinations. In order to reach maximum compliance of FXS subjects and their families in this exploratory biomarker study, a participant centric approach was chosen. The aim was to perform all clinical examinations including ERG and the CS test in a home setting with suitable tools and devices that allow for technical feasibility and low stress-inducing potential for the FXS subjects and care givers. The development of a portable ERG device by LKC for pediatric individuals was a great opportunity to use this device in the study since it avoids use of mydriatic eye drops, use of corneal electrodes and sedation. The RETeval® is a widely used medical device in clinical settings with appropriate regulatory clearances by the FDA and EMA in the USA (510(k) clearance) and EU (CE mark), respectively. It is ideally suited for the challenging behavior of FXS patients. The LEA SYMBOLS® low-contrast test is a clinically validated visual test [67–69] assessing contrast sensitivity developed for children as it offers a non-verbal/verbal response with no reading ability required. A further advantage of conducting the study in a home setting is that it allowed the investigators to adapt the flow of examinations to the abilities of each patient with an expanded time limit. The set-up of the experimental protocol allowed us to include and investigate FXS subjects as broadly as possible with a wide range of ID severity or autistic behavior. We were able to include 20 FXS subjects out of 21 initial volunteers for participation. An adequate ERG recording in at least one eye was successfully performed in 85% of subjects in the FXS cohort and 95% of age-matched healthy controls (95%). A complete CS evaluation was obtained for 80% of the FXS subjects but visual system evaluation using a combined ERG or CS approach provided evaluable data in 19 out of 20 FXS subjects or a 95% success rate. The ABC-CFX evaluation performed on the day of ophthalmic testing indicated that the cohort participating in this study were similar to typical subjects recruited in previously interventional studies [54, 70]. Also, the SSP results of the FXS cohort in this study had responses to common sensory events similar to other cohorts of disabled children such as those with ASD. The “definite-difference” categorization of the total SSP score suggests that the sensory processing in these FXS subjects were interfering with their functional performance and behavior. Overall, the biomarker-based approach used in this study appears to be practical and well-adapted and the study demonstrated that these evaluations can be done in individuals with intellectual disability like FXS.

The retina is composed of layers of specialized neurons that are interconnected through synapses [33, 72]. In the eye, light is captured by photoreceptor cells in the outer layer of the retina, which initiates a cascade of neuronal signals that reach the retinal ganglion cells whose axons form the optic nerve. These extend to the lateral geniculate nucleus in the thalamus and the superior colliculus in the midbrain, and then relay to the higher visual-processing centers [33]. Therefore, evaluation of retinal function by ERG provides insight into the initial stages of visual sensory processing [33]. In this study, ERG from FXS subjects showed a significant reduction in b-wave amplitude (37.6%) compared to control subjects. It is of note that the observed decrease in b-wave amplitude did not appear to be affected by subjects on psychotropic medications. Although this needs to be studied further to account for the low number of subjects in the psychotropic medication subset, the results hint that psychotropic medications may not be a substantial confound in the analysis of b-wave retinal physiology. If so, this would facilitate future studies in the recruitment of a broader population of subjects that includes those on psychotropic medications as part of their medical management. In this initial study, no significant change was observed in a-wave amplitude, which is derived principally from cones in the light-adapted response, and there was no change in the b:a ratio. The decreased b-wave amplitude implicates an alteration in bipolar cell signaling as these cells in this layer are mainly involved in b-wave genesis. This finding is further reinforced by the impaired LA flicker stimulation observed in FXS patients (27.5% decrease in flicker amplitude) and a 4.8% increase in flicker latency compared to control subjects in this study. Interestingly in support of these observations, comparing these data to a separate database from a cohort of healthy participants evaluated in the validation of the RETeval® device using identical recording methods and analysis parameters as used in this study (data kindly supplied by LKC) revealed non-overlapping and relatively broad differences confidence intervals between the mean a-wave amplitude, b-wave amplitude, and flicker amplitude in the FXS of this study and the REACT healthy control database (Table 1 Supplemental data). Alterations in flicker amplitude and/or latency implicate a sensitivity of the retinal signaling pathways to rapidly changing stimuli that stress pathways processing and communication that lead out of the retina. Alterations in bipolar cell layer function have been reported in *Fmr1*^*−/y*^ mice where a reduced b-wave amplitude by 24.4% has been observed [35, 40].

A significant reduction in a-wave amplitude was observed in the *Fmr1*^*−/y*^ mice [35, 40]. A trend in reduction of a-wave amplitude was also observed in this small FXS cohort and perhaps a study with a larger sample might be able to resolve a significant reduction in the cone response amplitude in the FXS phenotype. Although ERG recordings in mice used dark-adapted conditions and evoked a-wave responses from rods, bipolar cells still integrate rod or cone signals to produce the b-wave response. Thus, alterations in bipolar cell function appear to be similar in the retinal phenotype for both the *Fmr1*^*−/y*^ genetic mouse model of FXS and FXS individuals. Even if light conditions were different in the clinical biomarker trial (light-adapted) vs. the experimental conditions in *Fmr1*^*−/y*^ mice (dark-adapted), the commonality of the bipolar cell dysfunction is supported by the decreased flicker response in FXS patients, because this response is dominated by post-light receptor circuit elements, particularly the ON and OFF bipolar cells that interact to shape the steady-state flicker ERG response [73, 74]. Thus, the retinal alterations observed in *Fmr1*^*−/y*^ mice appear to translate to patients with FXS. Although molecular evidence of lack of FMRP protein in the retina of FXS individuals had not been reported, it can be speculated that bipolar cells and the inner retinal layers might present similar protein alterations and neuronal immaturity as observed in the FXS murine model [35, 40]. To our knowledge, this is the first time that a deficit in retinal function has been described with electrophysiology in individuals with FXS which corresponds to deficits previously observed in the *Fmr1*^*−/y*^ genetic mouse model of FXS.

The described alterations in retinal electrophysiology observed in FXS individuals could predict an effect on visual function. Contrast sensitivity represents a considerably more sensitive measure of altered retinal function than “standard” visual acuity measures [75]. CS changes are potentially more relevant than acuity, because alterations have direct behavioral consequences on the patient’s interaction and perception of their environment [67, 76, 77]. CS is defined by the threshold between the visible and invisible, and thus measured by the ability to detect subtle differences in shading, patterns, in detecting objects without clear outlines and discriminating objects or details from their background [78, 79]. Impairments of CS had been previously associated with retinal pathologies associated to visual function deficits [80–82]. In this study, the FXS group’s retinal electrophysiology alterations were associated with a higher contrast sensitivity threshold and lower performance (decreased by 41%) to discriminate several contrast intensities starting at 3 m of distance or greater. Using an optomotor device, *Fmr1*^*−/y*^ mice exhibit alterations in their visual skills, displaying a drop in their ability to understand a moving contrasted pattern, and a deficit in contrast discrimination [41]. Interestingly, the *Fmr1*^*−/y*^ mice phenotype were observed to have a lower ability to discriminate contrast by around 40% [41], and thus diminished CS seems to be an commonality in the endophenotype for both FXS and the animal model.

Therefore, both ERG and contrast perception alterations initially described in *Fmr1*^*−/y*^ mice have translational validity to humans with FXS. As supported in this study’s SSP evaluation and by the literature [15], FXS can produce alterations in the sensory processing that includes impairments in the visual system [21, 27, 29, 30]. Using eye-tracking, it was previously demonstrated that visual cerebral integration deficits in FXS led to misperception of contrast, texture, and moving stimuli [27, 29, 30, 83]. Our results are in alignment with deficits in spatiotemporal visual processing in FXS; however, we established for the first time that these alterations appear at least already at the level of the first stage of perception and integration of visual stimuli in the retina. Deficits in retinal function are thus critically involved in the visual sensorial FXS phenotype.

In summary, FXS patients exhibit visual function deficits as observed by pervasive impairments in motion perception and the ability to maintain the identity of dynamic object information during occlusion [20, 21, 27, 28, 30] and as demonstrated in the present study by deficits in contrast discrimination and retinal electrophysiology alterations. Such deficits in visual abilities have direct consequences on the development and performance of common tasks such locomotion control, gait, orientation, obstacles planning position [84–87], drawing skills [24, 25], or tasks involving manipulation of blocks to construct abstract designs [23–25], or requiring psychomotor coordination [23]. Interestingly, the deficits observed in our FXS cohort are in accordance with previous data showing an overall impairment of dorsal/ventral-stream circuits of vision processing [88–90]. Together, the data suggest that FXS features a selective deficit in the visual processing required for object description and tracking. Thus, these delayed sensory-motor features in FXS [22] might be linked to deficits in contrast discrimination. Indeed, CS alterations can affect the speed of visual processing [91] since a clear relationship was described between reaction times and increasing contrast level [92–94], as well as between visual evoked potentials and contrast gratings [94].

More importantly, these deficits may have an impact on social interaction and behavior as illustrated by the lower abilities of FXS patients in tasks assessing emotion recognition on faces. These tasks may reflect information processing and memory deficits rather than dysfunction in emotion-recognition [26]. The ability to recognize facial expressions and, therefore, gain socially relevant information is a fundamental requirement for normal reciprocal social interactions. The eyes are thought to be particularly important for understanding complex mental states [95] as well as the emotional state of others. Various studies associate this deficit in emotion recognition with reduced visual attention [96, 97] rather than an absence of recognition or reaction toward an emotion. Decoding facial characteristics or others’ emotions is a complex task beginning with the perception of sharp and discrete clues, as slight shadows, folding, and modifications in facial texture. Interestingly, these visual skills leading to lower abilities to emotion recognition could be part of the origin of the social anxiety of the pathology. There is broad evidence describing the link between social anxiety and emotion recognition problems as observed in many neuropsychiatric diseases such as schizophrenia [98], autism [99, 100] as well as FXS [97, 101]. A potential mechanism underlying this explanation is that individuals experiencing anxiety and social anxiety in particular, may view faces as a more threatening aspect of a social scene [102]. Avoidance of eye contact was clearly associated with social anxiety in both non-patient and social anxiety disorders [103]. Therefore, heightened looking to threatening stimuli may reflect hyper-vigilance for threatening stimuli, supporting previous literature indicating that socially anxious individuals fixate longer on the eye region of faces than those without social anxiety [104]. This potential explanation is supported by a previous eye-tracking study, which revealed a positive relationship between social dwell time on videos of actors approaching the viewer, and anxiety, in males with FXS [105]. All these alterations in sensory processing appear to be the core phenotype of the FXS pathology, as they cause impairment in processing and encoding of many types of sensory information, which may affect more complex social behaviors.

**Fig. 4:**
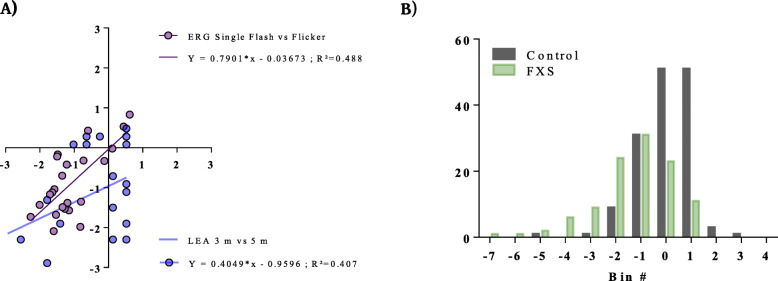
FXS subject *z*-score deviations from healthy control. **A** FXS subjects’ *z*-scores of single flash b-wave amplitude *vs* flicker amplitude ERG measures and *z*-scores of LEA SYMBOLS® low-contrast contrast sensitivity test measured at 3 m and 5% nominal contrast *vs* 5 m at 10% nominal contrast. Electrophysiological and functional visual system measures are consistent in FXS subjects. **B** Histogram of FXS subjects’ (green) and control subjects’ (black) *z*-scores for all single-flash b-wave amplitude and flicker amplitude ERG measures and LEA SYMBOLS® low-contrast contrast sensitivity test measured at 3 m and 5% nominal contrast *vs* 5 m at 10% nominal contrast. FXS subject scores are deviated overall to the left

FXS subjects’ *z*-score deviations from control were consistent for both ERG single flash b-wave amplitude and flicker amplitude measures as well as for contrast discrimination at 3 m and 5 m distances, indicating that these electrophysiological and visual functional measures were reliably measured and show some association in these FXS subjects (Fig. 4a). Together, ERG single flash b-wave amplitudes and flicker amplitudes as well as LEA SYMBOLS® low-contrast testing at 3 m and 5 m distances, serve to identify a FXS visual endophenotype. An examination of the *z*-scores for all data points comprising ERG single flash b-wave amplitudes and flicker amplitudes and LEA SYMBOLS® scores at 3 m and 5 m at nominal contrast levels of 5% and 10% respectively, one can see that overall there is about a − 1 standard deviation shift to the left for the cohort of FXS subjects when compared to the control group data for these tests (Fig. 4b). The distribution of *z*-scores also shows that some of these biomarker measures in FXS subjects were considerably different, up to 3–7 SD’s worse than the control group means. In considering potential therapeutic studies for improving retinal and visual system function, pre-identifying subjects with an endophenotype of moderate dysfunction appears to be possible. Given that both ABC-CFX and SSP scores indicated that this FXS cohort had behavioral and sensory processing differences from healthy populations, perhaps targeting a modest improvement in ERG and CS performance improvement might result in visual function benefits that are associated with behavioral improvements; however, further study is warranted.

Thus, improvement of sensory processing is one avenue for targeting core symptoms of the FXS phenotype, and ERG and CS are complementary electrophysiological and functional biomarkers that provide an avenue for facilitating therapeutic drug development in FXS and other conditions with retinal deficits in visual system function. Since the retina is a window to the brain [33], that results of our study suggest that a combined biomarker strategy involving ERG and CS (ERG-CS) offers a novel approach for investigating synaptic impairments in such diseases.

## Conclusions

Using ERG and CS, our investigation into the visual system of both mice and humans that genetically lack expression of FMRP (the pathogenic driver of FXS) has revealed the presence of sensory processing deficits involving reduce retinal function and poor visual contrast discrimination that are common to both [35, 40]. Consequences of such visual processing impairments are directly observable in the FXS clinical phenotypes, especially on social anxiety, social recognition, or learning difficulties. This demonstration is of a particular interest since we demonstrated the translatability and feasibility of assessing sensory abnormalities in the visual system of the *Fmr1*^-/*y*^ mice and FXS individuals with ERG and CS (ERG-CS). By combining complementary electrophysiological and functional readouts, the results of this study offer an objective, user-friendly, and readily measurable biomarker of visual sensory-processing dysfunction in FXS with potential application in related neurodevelopmental disorders that are currently being explored.

## Supplementary Information

**Additional file 1.**

## Data Availability

The data that support the findings of this study are available from Robert H. Ring (Kaerus Bioscience Ltd. CEO) but restrictions apply to the availability of these data, which were used under license for the current study, and so are not publicly available. Data are however available from the authors upon reasonable request and with permission of Robert H. Ring.

## Abbreviations

ABC-CFX: Aberrant Behavior Checklist–Community in FXS
ASD: Autism spectrum disorder
CNS: Central nervous system
CS: Contrasts sensitivity
ERG: Electroretinography
ERG-CS: Electroretinography-contrasts sensitivity
FXS: Fragile X syndrome
FMRP: Fragile X mental retardation protein
FMR1: Fragile X mental retardation gene 1
ID: Intellectual disability
LA: Light-adapted
ISCEV: International Society of Clinical Electrophysiology of Vision
SSP: Short Sensory Profile scale
Td: Troland unit
